# Supplementary material for: Glycoprotein Profile Measured by a 1H-Nuclear Magnetic Resonance Based on Approach in Patients with Diabetes: A New Robust Method to Assess Inflammation
Source: Life (Basel). 2021 Dec 16;11(12):1407. doi: 10.3390/life11121407 (PMC8709228; doi:10.3390/life11121407)
Supplement: Supplementary file 1 [file life-11-01407-s001.zip › life-1456760-supplementary.pdf]

life

MDPI

Online-Only Supplemental Material

Glycoprotein Profile Measured by a ^{1}H-Nuclear Magnetic Resonance Based on Approach in Patients with Diabetes: A New Robust Method to Assess Inflammation

Núria Amigó [1,2,3,*], Rocío Fuertes-Martín [1,2], Ana Irene Malo [3,4], Núria Plana [3,4], Daiana Ibarretxe [3,4], Josefa Girona [3,4], Xavier Correig [3,5] and Lluís Masana [3,4]

[1] Biosfer Teslab, Plaça del Prim 10, 2on 5a; 43201 Reus (Tarragona); Spain; rociofrtsm@gmail.com (R.F.-M.)
[2] Department of Basic Medical Sciences, Universitat Rovira i Virgili (URV), Institut d'Investigació Sanitària Pere Virgili (IISPV), Av. Universitat 1, 43204 Reus, Spain
[3] Centro de Investigación Biomédica en Red de Diabetes y Enfermedades Metabólicas Asociadas (CIBERDEM), Instituto de Salud Carlos III (ISCIII), Madrid, Spain; anairenemalo@gmail.com (A.I.M.); nuria.plana@salutsantjoan.cat (N.P.); daiana.ibarretxe@urv.cat (D.I.); josefa.girona@urv.cat (J.G.); xavier.correig@urv.cat (X.C.); luis.masana@urv.cat (L.M.)
[4] Vascular Medicine and Metabolism Unit, Research Unit on Lipids and Atherosclerosis, Sant Joan University Hospital, URV, IISPV, 43201 Reus, Spain
[5] Metabolomics Platform, Department of Electronic Engineering, URV, IISPV, 43007 Tarragona, Spain
* Correspondence: namigo@biosferteslab.com (N.A.); Tel:+34 676 12 99 66

Table S1. Covariance-adjusted linear regression models.

			**DEPENDENT VARIABLE**									
			**Area GlycB**		**Area GlycF**		**Area GlycA**		**H/W GlycB**		**H/W GlycA**	
			β	*p*	*β*	*P*	*β*	*p*	*β*	*p*	*β*	*p*
**INDEPENDENT VARIABLE**	**Age**	*Model A*	0.09	0.03 *	0.06	0.15	0.05	0.22	0.09	0.04 *	0.07	0.10
		Model B	0.02	0.58	-0.02	0.60	0.00	0.96	-0.03	0.46	-0.02	0.65
		Model C	0.02	0.61	-0.04	0.15	-0.02	0.37	-0.02	0.55	-0.03	0.32
	**Gender**	*Model A*	-0.02	0.64	0.00	0.86	0.02	0.56	-0.02	0.57	0.00	0.92
		Model B	-0.04	0.33	-0.03	0.41	0.00	0.87	-0.06	0.12	-0.03	0.43
		Model C	-0.08	0.04 *	-0.07	0.02 *	-0.04	0.05	-0.10	0.01 *	-0.07	0.02 *
	**BMI**	*Model A*	0.34	0.00 **	0.28	0.00 **	0.32	0.00 **	0.34	0.00 **	0.35	0.00 **
		Model B	0.26	0.00 **	0.19	0.00 **	0.26	0.00 **	0.21	0.00 **	0.26	0.00 **
		Model C	0.14	0.00 **	0.01	0.71	0.04	0.08	0.13	0.00 **	0.08	0.02 *
	**Diabetes**	*Model B*	0.20	0.00 **	0.27	0.00 **	0.17	0.00 **	0.37	0.00 **	0.28	0.00 **
		Model C	0.16	0.00 **	0.21	0.00 **	0.10	0.00 **	0.35	0.00 **	0.23	0.00 **
	**HDL-C**	*Model C*	-0.15	0.00 **	-0.06	0.11	-0.14	0.00 **	-0.17	0.00 **	-0.13	0.00 **
	**LDL-C**	*Model C*	0.06	0.10	0.09	0.00 **	0.10	0.00 **	0.04	0.33	0.13	0.00 **
	**TG**	*Model C*	0.35	0.00 **	0.67	0.00 **	0.75	0.00 **	0.22	0.01 *	0.5	0.00 **

The beta coefficients (β) and Anova p-value (p) are represented. Significance is marked (* for $p<0.05$ and ** for $p<0.01$). Abbreviations: BMI, body mass index; HDL-C, high density lipoprotein cholesterol; LDL-C, low density lipoprotein cholesterol; TG, total triglycerides; AHT, arterial hypertension; CVA, cerebral vascular accident.

Table S2. Two-way analysis of variance.

	(AD+) vs (AD-)	(DM+) vs (DM-)	(AD+) vs (DM+)
**Area GlycB**	0.00 **	0.01 *	0.59
**Area GlycF**	0.00 **	0.68	0.17
**Area GlycA**	0.00 **	0.30	0.02 *
**H/W GlycB**	0.00 **	0.00 *	0.02 *
**H/W GlycA**	0.00 **	0.00 **	0.00 **
**CRP**	0.02 *	0.00 **	0.27

p-values for a balanced two-way ANOVA. Significance is marked (* for $p<0.05$ and ** for $p<0.01$).
